# One-year comparative effectiveness of bupropion and nicotine replacement therapy in smoking cessation: A prospective observational cohort study in Turkey

**DOI:** 10.12669/pjms.42.4.13527

**Published:** 2026-04

**Authors:** Neslisah Gurel Koksal, Mustafa Koksal

**Affiliations:** 1 Neslisah Gurel Koksal, MD Department of Family Medicine, Giresun University Giresun Training, Research Hospital, Giresun, Turkey; 2 Mustafa Koksal, MD Giresun Provincial Directorate of Health, Giresun, Turkey

**Keywords:** Bupropion, Cohort study, Nicotine replacement therapy, Pharmacotherapy, Smoking cessation, Tobacco dependence

## Abstract

**Objective:**

Based on randomized clinical trials, bupropion and nicotine replacement therapy (NRT) are recommended as equally effective first-line pharmacotherapies for smoking cessation. However, long-term real-world observational data comparing these two treatment options are scarce. This study aimed to evaluate 12 months smoking cessation success rates among adult smokers attending a community health center–affiliated Smoking Cessation Clinic in Giresun, Turkey, comparing bupropion with nicotine replacement therapy (NRT) in a real-world clinical setting.

**Methodology:**

This prospective observational cohort study included 235 adult smokers who visited a single-center, community health center–affiliated Smoking Cessation Clinic in Giresun, Turkey, between October 2022 and October 2023. All participants received physician-led counseling and were prescribed either bupropion (n=45) or NRT (n=190; NRT 15 mg, n=118, and NRT 25 mg, n=72). Both treatments were provided free of charge for 12 weeks, during which six scheduled clinical visits were conducted over a 90-day period. Telephone follow-up assessments were conducted at weeks 16, 24 and 52. The primary outcome was the self-reported continuous abstinence at week 52.

**Results:**

The mean age of participants was 40.74 ± 12.42 years, with 59.6% (n=140) being men. The overall 12 months continuous abstinence rate was 69.4% (n=163). The independent predictors of sustained abstinence at 52 weeks were high adherence to treatment, regular attendance at scheduled follow-up visits, counseling along with bupropion or high-dose NRT (25 mg), and fewer adverse effects during treatment.

**Conclusion:**

In real-world clinical practice, bupropion or high-dose NRT (25 mg), combined with structured counseling yielded high long-term abstinence rates and acceptable side effect profiles.

## INTRODUCTION

Tobacco use remains a significant cause of morbidity and mortality worldwide and is strongly associated with cardiovascular, respiratory, and oncological diseases. According to the World Health Organization’s 2024 tobacco fact sheet, tobacco-related illnesses are responsible for approximately eight million deaths each year, making tobacco cessation a global public health priority.^1^

In Turkey, the prevalence of tobacco smoking has historically been high, and data from the Global Adult Tobacco Survey (GATS) 2016 indicate that approximately 32% (31.6%) of the adult population were current tobacco smokers in 2016,^2^ which directly contributes to an increased disease burden and mortality. However, in recent years, tobacco-control initiatives such as the Tobacco Control Program, implemented by the Turkish Ministry of Health, has notably increased public awareness and expanded structured smoking cessation services nationwide. Generally, individuals attending these outpatient clinics are evaluated by specialized physicians, who formulate personalized treatment plans according to their dependency levels and clinical characteristics, with regular follow-ups to support their smoking cessation process. Appointment and reminder systems are implemented to enhance patient adherence to treatment and attendance at clinical visits. Additionally, the “Alo 171 Smoking Cessation Counseling Hotline,” established by the Turkish Ministry of Health, provides round-the-clock (24/7) telephone counseling services, facilitates outpatient clinic appointments, and aims to strengthen individual motivation throughout the smoking cessation process.^3^

A retrospective study reported five-year abstinence rates of 22.5% for bupropion and 25.9% for nicotine replacement therapy (NRT), yet lacked detailed comparative analysis at the 12-month mark.^4^ Additionally, studies evaluating and comparing the efficacy of monotherapy and combination pharmacotherapy in real-world clinical settings in Turkey are lacking.

Nicotine dependence involves complex neurobiological mechanisms, including alterations in dopaminergic and noradrenergic pathways, complicating cessation attempts and elevating relapse risk.^5^ Systematic reviews and meta-analyses have demonstrated that pharmacotherapies, such as bupropion and NRT, significantly improve cessation rates compared to placebo or no treatment.^6^-^7^ Originally developed as an antidepressant, bupropion, a norepinephrine-dopamine reuptake inhibitor, has been approved as a first-line medication for smoking cessation. It reduces withdrawal symptoms and cravings by modulating mesolimbic dopaminergic pathways.^8^ Similarly, NRT, available in various formulations (patches, gums, and lozenges), reduces withdrawal symptoms by delivering controlled doses of nicotine. The effectiveness of NRT is well-documented, with studies indicating that dosage, duration, and mode of delivery influence cessation success.^9^

However, comparative long-term data on the real-world effectiveness of bupropion and NRT, particularly with structured follow-up protocols involving in-person visits and telephone follow-ups, remain limited. Therefore, to fill these gaps, we aimed to compare the one-year effectiveness of bupropion and NRT in a real-world clinical practice setting in Turkey. The purpose of this study was to provide critical real-world data that may be used to enhance ongoing tobacco control strategies and cessation interventions.

## METHODOLOGY

This prospective observational cohort study was conducted at the Smoking Cessation Clinic of Giresun Central Community Health Center.

### Ethical Approval:

The study protocol was approved by the Clinical Research Ethics Committee of Ordu University (approval Date: August 4, 2023; Decision No 2023/198), and all procedures were performed in accordance with the Declaration of Helsinki. Participant data were anonymized to ensure confidentiality, and informed consent was obtained from all participants prior to enrollment.

### Inclusion Criteria:

Between October 2022 and November 2023, a total of 312 patients applied to the Smoking Cessation Clinic seeking to quit smoking. Of these, 21 patients did not wish to continue the follow-up, and 56 either did not return to obtain the prescribed medications (provided free of charge at the community health center) or could not be reached owing to incorrect contact information. Thus, the study was completed with 235 participants, who were enrolled consecutively in the order of their clinical application and no randomization was performed.

### Exclusion criteria:

Individuals younger than 18 or older than 65 years; individuals with contraindications for pharmacological treatment (e.g., severe cardiovascular disease, uncontrolled hypertension, or a history of epilepsy); refusal to participate in the study; individuals diagnosed with serious psychiatric disorders such as schizophrenia, bipolar disorder, or psychotic disorders; individuals unable to communicate or incapable of understanding the scope of the study due to mental retardation or cognitive impairment; those who could not be contacted or followed up due to missing or incorrect contact information; pregnant or breastfeeding women; individuals who had received smoking cessation treatment within the last three months; individuals who had participated or were participating in another clinical trial within one month; and individuals with alcohol or substance dependence. The exclusion criteria were established to ensure the reliability and external validity of the research findings.

At the baseline evaluation, data were collected on the participants’ age, sex, educational level, employment status, age at smoking initiation, comorbid conditions, number of cigarettes smoked daily, total duration of smoking (in years), reasons for wanting to quit, and the treatment methods applied. Smoking behavior and previous quitting attempts were assessed face-to-face using a questionnaire developed by the researchers based on the current literature. Nicotine dependence levels were measured using the Fagerstrom Test for Nicotine Dependence (FTND), a six-item instrument validated in Turkish language. Patients were classified according to their FTND scores into low (FTND score 0-3), moderate (4-6), and high (7-10) nicotine dependence categories.^10^

### Follow-up procedures:

At the initial clinical evaluation, patients were assigned to one of the three different pharmacological treatment groups based on their FTND scores, comorbidities, and availability of free treatment options at the time of their visit. The treatment modalities used in this cohort were bupropion and NRT.

All pharmacological treatments were provided free of charge through the Republic of Turkey Ministry of Health’s National Tobacco Addiction Treatment and Support System. In addition, at each clinic visit, the participants received behavioral counseling from the physician for smoking cessation. Behavioral counseling was delivered face-to-face by the clinic physician at each scheduled visit and was tailored to the participant’s level of nicotine dependence, previous quit attempts, and reported barriers to cessation. At the baseline session, counseling included: brief assessment of smoking patterns, motivation to quit, and prior cessation experiences; clear, personalized advice to quit and discussion of expected health benefits; collaborative development of an individualized quit plan (including identification of high-risk situations/triggers and coping strategies); education about nicotine withdrawal symptoms and craving management techniques; and instruction on correct use of the prescribed pharmacotherapy and the importance of adherence. Each treatment program lasted 12 weeks, and six clinic visits were scheduled within a 4-month period on days 7, 14, 30, 60, 90, and 120 of treatment to enhance follow-up and compliance. At every follow-up visit, the participants’ adherence to the treatment, any side effects experienced, and their smoking status were systematically recorded, and further counseling was provided as needed. Participants were contacted over telephone 24 and 52 weeks after the start of treatment to assess their smoking status at those time points.

To evaluate the treatment outcomes, sustained smoking cessation was defined as the participant’s self-reported continuous abstinence from smoking for the entire 12-month period following treatment initiation. Participants who initially quit but then resumed smoking at any point during follow-up were considered to have experienced a relapse.

### Statistical Analysis:

All statistical analyses were performed using NCSS 2020 Statistical Software (NCSS LLC, Kaysville, Utah, USA). Descriptive statistics were used to summarize the data. Quantitative variables were presented as mean, standard deviation, median, minimum, and maximum values, and qualitative variables were presented as frequencies and percentages. The conformity of quantitative data to a normal distribution was evaluated using the Shapiro-Wilk test and Box Plot graphs.

For comparisons between two groups, Student’s t-test was used if the data were normally distributed, whereas the Mann-Whitney U test was used for non-normally distributed variables. Comparison of categorical (qualitative) data was done using Pearson’s chi-square test or Fisher’s exact test, as appropriate. Results were interpreted within a 95% confidence interval, and a p-value of < 0.05 was considered statistically significant.

## RESULTS

The study involved 235 participants, of whom 59.6% (n=140) were men. The detailed demographic characteristics of the participants are presented in Table-I and Table-II shows comparison of their characteristics based on relapse status.

**Table-I:** Distribution of Descriptive Characteristics.

Characteristic	n (%) or Mean ± SD
***Gender***	
Male	140 (59.6)
Female	95 (40.4)
***Age (years)***	
Mean ± SD	40.74 ± 12.42
Median (Range)	41 (19-65)
***Education Level***	
Less than High School	85 (36.2)
High School or above	150 (63.8)
***Employment Status***	
Student	29 (12.3)
Employed	111 (47.2)
Unemployed	48 (20.4)
Retired	47 (20.0)
***Age at Smoking Initiation***	
<18 years	76 (32.3)
≥18 years	159 (67.7)
***Comorbidities***	
None	96 (40.9)
Present	139 (59.1)
Diabetes	31 (13.2)
Cardiovascular Disease	49 (20.9)
Chronic Obstructive Pulmonary Disease (COPD)	26 (11.1)
Cancer	2 (0.9)
Psychiatric Disorders	6 (2.6)
Other	28 (11.9)
***Daily Cigarette Consumption at Baseline***	
<20 cigarettes/day (Light smoker)	119 (50.6)
≥20 cigarettes/day (Heavy smoker)	116 (49.4)
***Total Duration of Smoking***	
<10 years	58 (24.7)
10-20 years	88 (37.4)
>20 years	89 (37.9)

***Note:*** Participants may have more than one comorbid condition. SD, standard deviation.

**Table-II:** Comparison of Participant Characteristics According to Relapse Status.

Characteristics	No Relapse (n=163) n (%)	Relapse (n=72) n (%)	p-value
***Gender***			0.158^a^
Male	102 (62.6)	38 (52.8)	
Female	61 (37.4)	34 (47.2)	
***Age (years)***			0.338^b^
Mean ± SD	41.20 ± 12.49	39.68 ± 12.27	
Median (Min-Max)	41 (19-65)	38 (20-64)	
***Educational status***			0.234^a^
Below high school	63 (38.7)	22 (30.6)	
High school or above	100 (61.3)	50 (69.4)	
***Employment status***			0.695^a^
Student	19 (11.7)	10 (13.9)	
Employed	74 (45.4)	37 (51.4)	
Unemployed	35 (21.5)	13 (18.1)	
Retired	35 (21.5)	12 (16.7)	
***Age at smoking initiation***			0.931^a^
<18 years	53 (32.5)	23 (31.9)	
≥18 years	110 (67.5)	49 (68.1)	
***Presence of comorbidities***			0.456^a^
No	64 (39.3)	32 (44.4)	
Yes	99 (60.7)	40 (55.6)	
***Number of cigarettes/day at baseline***			0.878^a^
<20/day	82 (50.3)	37 (51.4)	
≥20/day	81 (49.7)	35 (48.6)	
***Total duration of smoking***			0.713^a^
<10 years	38 (23.3)	20 (27.8)	
10-20 years	61 (37.4)	27 (37.5)	
>20 years	64 (39.3)	25 (34.7)	

a Pearson’s Chi-square test;

b Student’s t-test SD, standard deviation.

The participants’ nicotine dependence scores ranged from 1 to 10, with a mean FTND score of 7.22±2.60. While 60% (n=141) of the participants reported no previous smoking cessation attempts, 40% (n=94) reported at least one prior attempt. Results revealed that fear of illness was the most frequently cited motivation (71.5%, n=168) for quitting smoking, followed by physician recommendations (42.6%, n=100), economic reasons (33.2%, n=78), close relatives quitting smoking (29.4%, n=69), social exclusion (26.4%, n=62), and existing health problems (25.5%, n=60).

The most frequently prescribed smoking cessation therapy was counseling combined with 15 mg NRT, used by 50.2% (n=118) of the participants, followed by counseling combined with 25 mg NRT (30.6%, n=72), and counseling with bupropion (19.1%, n=45) (Table-III).

**Table-III:** Comparison of Smoking Cessation Treatments According to Relapse Status.

Smoking cessation treatment	No Relapse (n=163) n (%)	Relapse (n=72) n (%)	p-value
Counseling + Bupropion	36 (22.1)	9 (12.5)	0.009**
Counseling + 15 mg NRT	71 (43.6)	47 (65.3)	
Counseling + 25 mg NRT	56 (34.4)	16 (22.2)	

***Abbreviation:*** NRT, nicotine replacement therapy; a Pearson’s Chi-Square test. *Statistically significant (p < 0.01).

The most commonly reported adverse effects during treatment were stomach pain and nausea (17.9%, n=42), sleep disturbances (16.2%, n=38), dizziness and light-headedness (8.1%, n=19), skin lesions (8.1%, n=19), palpitations and chest pain (4.7%, n=11), vivid dreams and hallucinations (3.4%, n=8), and other adverse effects (1.7%, n=4).

At the end of the one-year follow-up period, 69.4% (n=163) of the participants had successfully quit smoking without relapse, whereas 30.6% (n=72) had resumed smoking. Finally, the factors predicting sustained smoking cessation at 52 weeks included high adherence to treatment, regular attendance at scheduled clinical follow-up visits, receiving counseling combined with either bupropion or high-dose NRT (25 mg), and experiencing fewer adverse effects during treatment.

## DISCUSSION

This study demonstrated the substantial efficacy of combining counseling and pharmacological therapies for smoking cessation. This finding aligns with previous literature, emphasizing the role of structured interventions in supporting long-term smoking cessation success.^11^ Of particular note was the high cessation rate of 69.4% observed at the one-year follow-up visit. The clinic’s accessible location and patient population’s preference for pharmacological agents rather than counseling alone may have contributed to these high success rates.

In the current study, 52.8% of relapses occurred among male patients; however, no statistically significant difference was found between the sexes in smoking cessation rates (p > 0.05). This finding contrasts with studies indicating gender-based variability in cessation success,^12^,^13^ but aligns with research suggesting no significant impact.^14^ These discrepancies imply that sex-related differences in cessation outcomes may be influenced by sociocultural factors, psychological states, and treatment methods. Therefore, further studies with larger and more heterogeneous samples are necessary to elucidate sex-based smoking cessation outcomes.

The average FTND score measured in our study was 7.22, indicating high nicotine dependence among participants, which complicates sustained smoking cessation. For instance, in the ATTEMPT cohort study individuals with high FTND scores had significantly increased relapse risk compared to those with lower dependence scores.^15^ However, our study did not identify a significant relationship between the FTND scores and relapse rates which may be attributed to the relatively homogeneous dependence levels among participants, the intensive behavioral counseling provided to all patients irrespective of dependence severity, and the potential efficacy of high-dose pharmacotherapies. Additionally, our sample size may have limited the statistical power to accurately detect this relationship.

Motivational factors such as fear of illness, physician recommendations, successful smoking cessation through close contact, and the presence of chronic diseases were frequently reported as reasons encouraging participants to quit smoking. The presence of chronic diseases or fear of illness are identified as potent motivators for smoking cessation.^16^ However, in our study, the presence of chronic disease did not significantly influence cessation success. This may be related to variations in patients’ perceived severity of their illness, high levels of nicotine dependence, psychological factors, and use of smoking as a stress management strategy. Thus, interventions focusing solely on presence of disease may not suffice, indicating the necessity for personalized behavioral and psychological interventions for individuals with chronic conditions.

Successful smoking cessation of a close contact was identified as a significant motivating factor in our study. This aligns with previous research demonstrating that partner-focused interventions enhance behavioral change.^17^ Additionally, economic factors are significant motivatorsas there was increased intention to quit smoking following cigarette price increase, particularly in low-to middle-income groups.^18^ Given that only 47.2% of the participants in our study were employed, economic factors appear to be critical in motivating cessation attempts.

Physician recommendations were cited by 42.6% of participants as influential in their decisions to quit smoking. Personalized recommendations from healthcare providers on initiating and sustaining smoking cessation attempts have a positive impact.^19^ However, no significant impact was observed in our study. This may be due to diminishing initial motivation over time, behavioral resistance due to high nicotine dependence, insufficient personalized and continuous behavioral support, or inconsistencies in the follow-up processes. Hence, reinforcing long-term personalized psychological and social support along with physician recommendations is crucial for enhancing sustained cessation success in clinical practice.

In our study, relapsed participants frequently used counseling combined with 15 mg of NRT. This outcome might be related to the prevalent provision of 15 mg NRT through the National Tobacco Addiction Treatment and Support System. However, low- or moderate-dose NRT may be inadequate for individuals with high nicotine dependence, potentially reducing cessation success and increasing relapse risk.^7^,^9^,^20^ Therefore, initiating treatment with high-dose NRT (e.g., 25 mg) or combined pharmacotherapies may enhance long-term cessation outcomes in these individuals. Policy planning should ensure the consistent availability of varied NRT doses, particularly for highly dependent populations.

A notable finding of our study was the 80% cessation rate among participants receiving counseling combined with bupropion, known to enhance cessation attempts by inhibiting dopamine and norepinephrine reuptake, thereby reducing withdrawal symptoms and cigarette cravings.^6^,^21^ Furthermore, the mild antidepressant effects of bupropion mitigate negative mood and stress and enhance psychological resilience during cessation attempts.^22^ Therefore, our results strongly support the use of bupropion in highly dependent patients.

The observed cessation rate (69.4%) aligns closely with that of previous systematic reviews and randomized controlled trials that evaluated similar pharmacological treatments. For instance, bupropion increased cessation likelihood by 60% compared to that by placebo (Relative risk=1.60; 95% Confidence interval=1.49-1.72), with a long-term cessation rate around 20%.^21^ Comparable results have been reported in network meta-analyses and systematic reviews.^7^,^23^

The most frequently reported adverse events were nausea, insomnia, and dizziness, consistent with those reported previously.^23^-^25^ Informing patients about these potential mild side effects is crucial for enhancing medication adherence. Furthermore, the incidence of side effects was significantly higher among relapsed participants (p=0.003), indicating that effective management of side effects may reduce the risk of relapse.

### Strengths of this study:

The key strengths of this study include its prospective observational cohort design, detailed initial assessments, structured clinical follow-up, and relevance to real-world clinical practice within the Republic of Turkey Ministry of Health’s National Tobacco Addiction Treatment and Support System.

### Limitations:

Several limitations should be considered when interpreting these findings. This was a single-center study conducted in a community health center–affiliated smoking cessation clinic in Giresun, Turkey, and the sample comprised smokers who actively sought clinical support; therefore, cessation rates and treatment patterns may not be fully generalizable to smokers who do not attend cessation clinics or to regions with different healthcare access, socioeconomic characteristics, and sociocultural norms. Although counseling followed a standardized clinical framework, unmeasured local sociocultural and contextual factors (e.g., social norms, household/peer influences, and acceptability of physician-led counseling) may have affected engagement and outcomes. Smoking status relied on self-report without biochemical verification, and some follow-up assessments were conducted by telephone, which may introduce recall and social desirability bias. In addition, as a non-randomized observational study, treatment allocation depended on clinical factors (FTND score, comorbidities) and the availability of free pharmacotherapy, raising the possibility of confounding by indication; unequal group sizes may also have limited comparisons. Finally, excluding individuals using psychiatric medications limits applicability to smokers with psychiatric comorbidities. However, limitations such as reliance on self-reported smoking status without biochemical verification and the inconsistent availability of free pharmacological treatments throughout the year might have affected the reported cessation rates. Additionally, excluding psychiatric medication users limits the generalizability of the results to patients with psychiatric comorbidities.

## CONCLUSION

This study demonstrated that under real-world clinical conditions, combined pharmacological therapies (bupropion and high-dose nicotine replacement therapy) and behavioral counseling are highly effective in achieving long-term smoking cessation. Factors such as high adherence to treatment, regular attendance of scheduled clinical visits, and effective management of treatment-related side effects were identified as crucial determinants of sustained abstinence. Our findings support the effectiveness of structured smoking cessation services provided by the Turkish national healthcare system, emphasizing the importance of expanding these interventions to a broader population. However, owing to methodological limitations, further large-scale, long-term studies employing objective biochemical verification methods are warranted to confirm and extend these results.
